# Transient Non-Native Helix Formation during the Folding of β-Lactoglobulin

**DOI:** 10.3390/biom4010202

**Published:** 2014-02-13

**Authors:** Masamichi Ikeguchi

**Affiliations:** Department of Bioinformatics, Soka University, 1-236 Tangi-cho, Hachioji, Tokyo 192-8577, Japan; E-Mail: ikeguchi@soka.ac.jp; Tel.: +81-426-91-9444; Fax: +81-426-91-9312

**Keywords:** α-helix, β-sheet, stopped-flow, circular dichroism, hydrogen-deuterium exchange, mutant protein

## Abstract

In ideal proteins, only native interactions are stabilized step-by-step in a smooth funnel-like energy landscape. In real proteins, however, the transient formation of non-native structures is frequently observed. In this review, the transient formation of non-native structures is described using the non-native helix formation during the folding of β-lactoglobulin as a prominent example. Although β-lactoglobulin is a predominantly β-sheet protein, it has been shown to form non-native helices during the early stage of folding. The location of non-native helices, their stabilization mechanism, and their role in the folding reaction are discussed.

## 1. Introduction

Theoretical studies have shown that short-range interactions and long-range interactions are consistent with each other in ideal proteins. This is known as the consistency principle [1] or the principle of minimum frustration [2]. Of course, the consistency is not perfect in real proteins, and many reports have shown that the non-native structure is formed transiently during protein folding [3,4,5]. Such non-native interactions or structures have been attracting attention because they may be related to misfolding and diseases [6].

**Figure 1:**
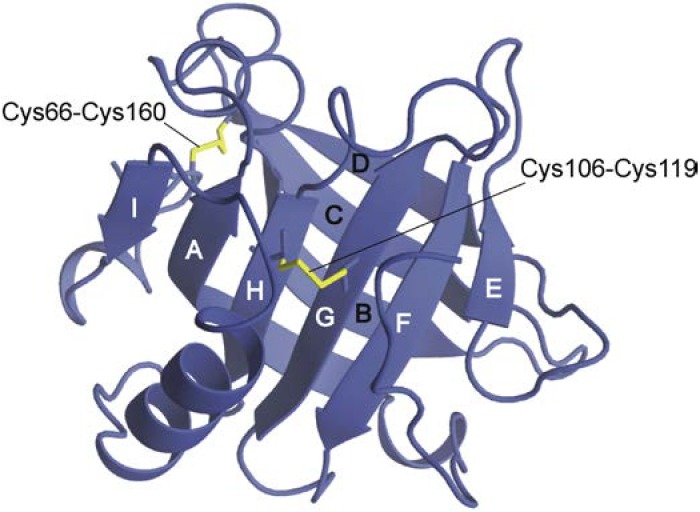
Schematic representation of the three-dimensional structure of β-lactoglobulin. The structure was illustrated on the basis of Protein Data Bank (PDB) coordinates (1BSY [8]) using MolFeat (FiatLux, Tokyo, Japan). Strands are labeled A–I, and disulfide bonds are shown in yellow.

A prominent example of the formation of non-native structures was observed during the folding of β-lactoglobulin. This protein has an eight-stranded (termed A–H) β-barrel structure that is flanked by a major helix and the off-barrel strand I (Figure 1). The first indication of non-native helix formation was obtained by Kuwajima and co-workers [7], who developed a stopped-flow circular dichroism (CD) apparatus and followed CD changes during the folding reactions of cytochrome *c* and bovine β-lactoglobulin (BLG). They observed that far-UV CD signals appeared mainly within the dead time of measurements (18 ms), whereas near-UV CD signals developed within the resolvable time range (from 0.1 to 500 s). From these observations, those authors concluded that the formation of both the α-helix and β-sheet precedes the acquisition of rigid tertiary structures. Although the authors recognized that the far-UV CD signal of BLG is much more negative than is the value of the native conformation, they did not mention the possibility of non-native helix formation in that paper.

Later, Goto’s group performed similar experiments and concluded that non-native α-helices were formed within the dead time of their stopped-flow experiment (10 ms) [9]. Before these experiments, they provided several circumstantial lines of evidence in support of their conclusion. First, the secondary structure of BLG was predicted to be mainly α-helical based on its amino acid sequence [10]. Second, BLG assumed a highly helical conformation in the presence of trifluoroethanol (TFE), which induces an α-helical conformation, whereas another β-sheet protein, the C_L_ fragment of immunoglobulin, did not show a helical conformation, even in the presence of TFE [10]. Third, peptide fragments of BLG showed helicity, even though they corresponded to the β-strand regions of the native structure of BLG (see Table 1) [11,12].

Kuwajima’s group also reinvestigated the folding kinetics of BLG using stopped-flow X-ray scattering, in addition to UV absorption, fluorescence, and CD spectroscopy [13,14]. Those studies revealed that the burst-phase intermediate with non-native α-helices had a radius of gyration that was close to that of the native state. In collaboration with Kuwajima’s group, we also showed the overshoot of the burst-phase CD for equine β-lactoglobulin (ELG) [15]. ELG is monomeric and has no free cysteine residue, whereas BLG is dimeric at neutral pH and has a free cysteine residue. Therefore, experiments pertaining to BLG were restricted to an acidic pH, to prevent complications stemming from dimer formation and disulfide interchange. Because ELG is free from such restrictions, we used ELG rather than BLG in subsequent studies.

## 2. Equilibrium Intermediates

To characterize non-native structures in detail, stable analogues of the burst-phase intermediate are quite useful because various techniques, such as NMR, can be used to obtain these structures. Hamada and Goto [16] used a low concentration of TFE to increase the population of a helical intermediate during equilibrium unfolding induced by guanidine hydrochloride (GdnHCl). Although the population of the intermediate was actually increased, it coexisted with the native and unfolded conformations, which hampered the structural characterization of the intermediate. Kuwata *et al.* [17] assigned ^1^H, ^13^C, and ^15^N chemical shifts of TFE-denatured BLG, and helical regions were identified from secondary shifts. Although 10–14 segments assumed helical conformations, it was not clear whether the helical region that formed in TFE was also helical in water. Although Kuwata *et al*. [18] also investigated the pressure-induced unfolding of BLG by using heteronuclear two-dimensional NMR and showed that a variety of partially unfolded conformations had accumulated, the location of non-native helices was not clarified. Katou *et al.* [19] characterized the cold-denatured state of BLG. BLG loses its tertiary structure at 0 °C in the presence of 4 M urea, but retains some secondary structures, as indicated by CD spectra. Small-angle X-ray scattering (SAXS) experiments showed that the radius of gyration was 25 Å, which is larger than the value observed in the native state (20 Å), but much smaller than the value observed in the unfolded state (37 Å). The H/D exchange experiments provided a residue-specific view of the cold-denatured conformation. Strong protection against H/D exchange was observed for the residues located in strands G and H, and the authors concluded that a native-like β-hairpin was retained in the cold-denatured state. Conversely, CD spectra as well as amide protection showed no indication of non-native α-helices for the cold-denatured state.

**Figure 2:**
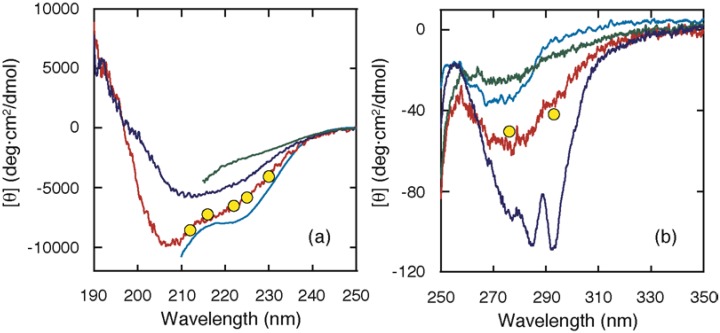
Circular dichroism (CD) spectra of the N, A, C, and U states, and the burst-phase CD values in far **(a)** and near **(b)** UV regions. The spectra of the N (blue) and A (red) states were acquired at pH 7.0 and 1.5, respectively, and at 25 °C [20]. The spectrum of the C state (cyan) was measured in the presence of 2 M urea at pH 4.0 and –10 °C [21]. The spectrum of the U state (green) was measured in the presence of 8 M urea at pH 4.0 and 25 °C. The CD values attained within the burst phase of the folding kinetics at pH 4.0 and 25 °C are shown by yellow circles [15].

In contrast, ELG exhibited a stable intermediate (A state) at acid pH or at moderate denaturant concentrations, which provided an opportunity to characterize the structure of the intermediate in detail [15,20]. It is important that the A state be indistinguishable from the kinetic intermediate that accumulates within the burst phase. Figure 2 shows the CD spectra of the native (N), acid (A), and unfolded (U) states and the burst-phase values attained within the dead time. The burst-phase values coincided well with the spectrum of the A state, indicating that the conformation of the burst-phase intermediate is similar to that of the A state. The conformation of the A state has been characterized at equilibrium [20]: (1) the A state has a substantial secondary structure, as indicated by the far-UV CD spectrum (Figure 2); (2) it lacks the rigid tertiary packing of the side chains, as shown by elimination of the near-UV CD intensity (Figure 2) and of the wide dispersion of the chemical shifts; (3) it is nearly as compact as the N state, as shown by gel-filtration and sedimentation experiments; and (4) it has an exposed hydrophobic surface, as indicated by its tendency to aggregate. All of these characteristics imply that the A state is a molten globule state, although its secondary structure contains non-native α-helices.

The cold-denatured (C) state of ELG exhibited a unique structural characteristic [21]: its CD intensity was much higher than that of the A state (Figure 2), indicating that a larger amount of non-native helices are formed in the C state. This is a striking difference from the cold-denatured state of BLG. Although secondary structures including non-native helices are formed in the C state, SAXS showed that the radius of gyration is 37 Å, which is much larger than the values observed in the N state (19 Å) and in the A state (22 Å), but is similar to the value observed in the U state (40 Å). Because the scattering curve itself is similar to that of the unfolded state, the C state is considered to have a chain-like conformation with many helical stretches.

## 3. Location of Non-Native Helices

### 3.1. H/D Exchange

**Figure 3:**
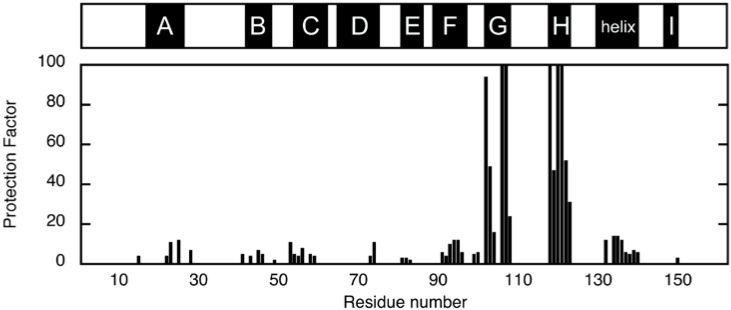
Protection factors of backbone amide protons in the A state at pD 1.5 and 25 °C [22]. The upper bar indicates the location of the secondary-structure units in the N state.

The location of non-native helices was first investigated by H/D exchange experiments, in which the amide proton is exchanged with deuteron when the protein is placed in D_2_O. This exchange is retarded if the amide proton establishes a hydrogen bond or is buried inside the protein. The degree of retardation is usually expressed as a protection factor *P*, which is given as a ratio of the exchange rate of the unstructured amide proton and the observed exchange rate. Figure 3 shows the protection factors of individual residues of ELG in the A state, which were calculated from the exchange rate determined from the peak-volume decrease observed in the two-dimensional NMR [22]. Strong protections were observed for residues located in the G and H strands. Because weak protections were found in the A and F strands, which are the hydrogen-bonding partners of the H and G strands in the N state, respectively, these data were first interpreted as an indication of the formation of a native-like β-sheet in the A state [22]. Weak but contiguous protections were found for the residues located in the major C-terminal helix in the N state, which suggest that this helix is also formed in the A state. Similar patterns of protection were observed for the cold-denatured state [19] and a kinetic intermediate of BLG [23], and were also interpreted as evidence of the formation of a native-like β-sheet.

### 3.2. Proline-Scanning Mutagenesis

**Figure 4:**
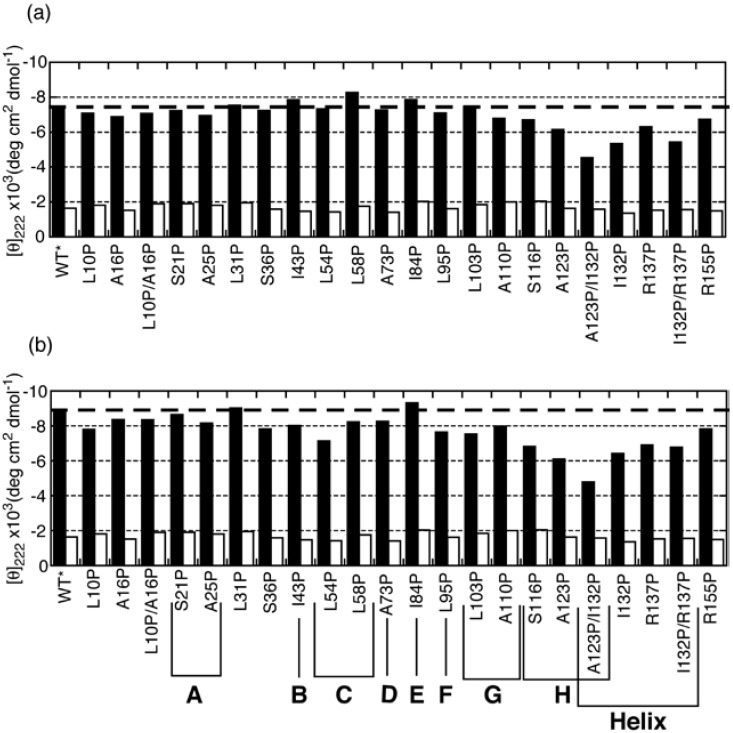
[θ]_222_ in the A, C, and U states of pseudo-wild-type (WT*) and its proline mutants. **(a)** [θ]_222_ for WT* and all proline-substituted mutants in the A state (solid bars) and in 6 M GdnHCl (open bars). **(b)** [θ]_222_ for WT* and all proline-substituted mutants in the C state (solid bars) and in 6 M GdnHCl (open bars). The dashed line represents [θ]_222_ for WT*. The secondary-structure elements in which the proline-substituted residues are included are shown in the bottom panel **(b)**. Adapted with permission from reference [24]. Copyright 2006 American Chemical Society.

Although the H/D exchange experiments revealed the structured region in partially folded intermediates, it is difficult from such data to determine the type of secondary structure, *i.e*., whether an α-helix or a β-sheet is formed. To overcome this difficulty, we performed a proline-scanning mutagenesis experiment [24]. This method is based on the concept that the CD spectrum will change if a proline is substituted for a residue that is involved in an element of secondary structure. It is possible to distinguish between α-helices and β-sheets because prolines destabilize both α-helical and β-sheeted structures and because these two types of structures exhibit different CD spectral changes when they are disrupted. In this experiment, we used a single disulfide mutant in which Cys66 and Cys160 were substituted with alanine (C66A/C160A) as a pseudo-wild-type (WT*) ELG for proline substitution. It is known that WT* produces CD spectra that are similar to those of WT under conditions in which the native and molten globule states are stabilized [25]. WT* also assumes the C state at low pH and low ionic strength, even at 25 °C [25]. In Figure 4, the mean residue ellipticity at 222 nm ([θ]_222_) is compared between WT* and proline mutants under the A-state conditions (0.1 M HCl–KCl, pH 1.5), the C-state conditions (0.1 M phosphoric acid), and the U-state conditions (6M GdnHCl). 

**Figure 5:**
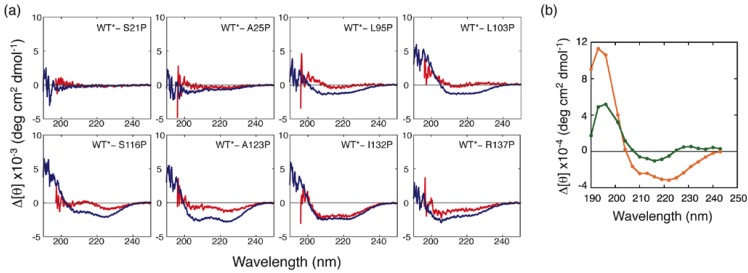
**(a)** Differences in CD spectra between the mutants and WT* in the A and C states (shown in red and blue, respectively) and **(b)** differences in CD spectra between α-helix and unordered form (orange) and between β-sheet and unordered form (green) [26].

Under the A-state conditions, a remarkable increase in [θ]_222_ (reduction in bar height in Figure 4) was observed for mutants of the residues located in the H strand and in the C-terminal helix. This indicates that a non-native helix is formed in the H-strand region, and a native-like helix including I132 and R137 is also formed in the A state. Although mutation of residues located in the F and G strands (L95P and L103P) did not lead to an increase in [θ]_222_, these residues yielded significant protection against H/D exchange (Figure 3). This apparent discrepancy was resolved by inspecting the differences in CD spectra between the mutants and WT* (Figure 5). Although the [θ]_222_ values of L95P and L103P are nearly identical to that of WT*, the difference spectra showed a weak negative peak around 215 nm and a positive difference below 210 nm (Figure 5(a)). These characteristics are also observed in the difference spectrum observed between the reference spectra for β-sheet and unordered form (Figure 5(b)), indicating that L95 and L103 form a β-sheet. Although Kuwata *et al*. observed weak protection against H/D exchange for residues 12–21 of BLG, and concluded that this region assumes a non-native helix [23], the proline-scanning mutagenesis experiments did not detect a stable helix in this region.

In the C state, L95P and L103P, in addition to the residues located in the H strand and in the C-terminal helix, yielded a significant increase in [θ]_222_ (Figure 4(b)). The difference in CD spectra showed that these residues form helical conformations (Figure 5(a)). Furthermore, other residues, with the exception of S21, L31, and I84, yielded a significant increase in [θ]_222_ (Figure 4(b)). These results, combined with SAXS findings, suggest that the C state is an ensemble of conformations in which helical stretches are formed at various locations in a nearly random coil polypeptide.

### 3.3. NMR Analysis of a Fragment

Although proline-scanning mutagenesis revealed that a non-native helix is formed at a region including A123, the length of the helix remained unknown. To identify the helical region in the A state, we tried to measure the NMR spectrum of ELG in the A state. Because ELG in the A state tends to aggregate, the ^1^H NMR spectrum was acquired at a low protein concentration within a short period after dissolving the lyophilized sample in 0.1 M HCl–KCl (pH 1.5) [20]. The chemical shift dispersion, which is typical of globular proteins, was largely lost in the NMR spectrum of the A state, indicating that the rigid tertiary packing of the side chains was absent in the A state. Multidimensional spectrum measurements, which are required for resonance assignment, were not allowed because of aggregation during a long measurement.

**Figure 6:**
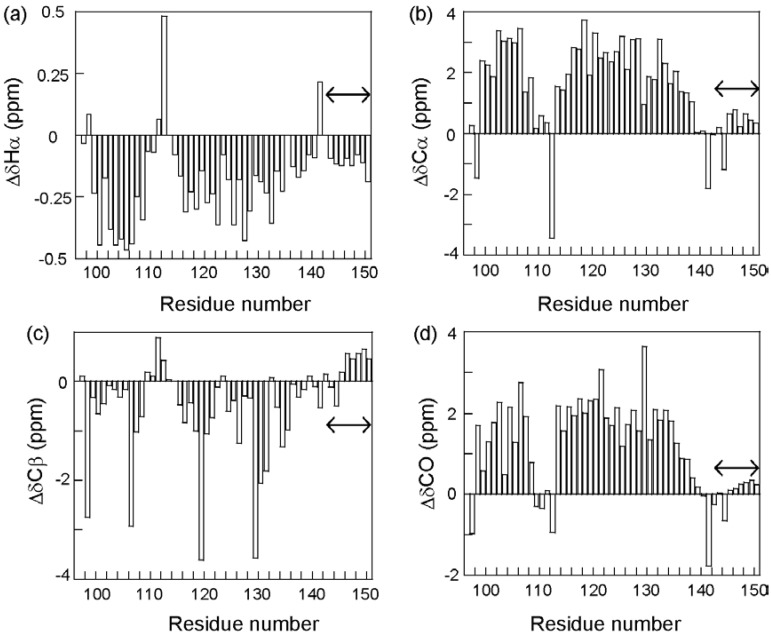
Secondary shifts of ^1^Hα **(a)**, ^13^Cα **(b)**, ^13^Cβ **(c)**, and ^13^CO **(d)** resonances of CHIBLΔF. The residue numbers are given as the corresponding number in the full-length protein. Double arrows indicate the hexahistidine-tag region.

To circumvent this difficulty, we looked for ELG fragments in which the structure found in the full-length protein was maintained. Fortunately, a fragment corresponding to residues 88–142 assumed a mainly helical structure that was similar to the C state of the full-length protein [27]. This fragment was termed “core of the helical intermediate of β-lactoglobulin” (CHIBL). A shorter fragment, CHIBLΔF (residues 97–142), also assumed a similar helical structure and was soluble, thus allowing the acquisition of three-dimensional NMR spectra. Thus, the complete backbone resonance assignment was accomplished for ^13^C- and ^15^N-labeled CHIBLΔF [28]. It is well known that the resonances of Hα and Cβ shift downfield, whereas those of Cα and CO shift upfield when they are included in a helical conformation [29]. Figure 6 shows the secondary shifts (differences from the random coil shifts) for residues of CHIBLΔF, which indicate that non-native α-helices were formed by residues 98–107 and 114–135. Interestingly, a non-native helix at the H-strand region and a native-like C-terminal helix merged into a long helix.

In conclusion, the non-native helices are formed at various sites, including residues 98–107 and 114–135, in the C state. In the A state, residues 114–135 probably assume a non-native helix, but L95 and L103 seem to be involved in a native-like β-hairpin. The earlier interpretation of H/D exchange data, in which the G and H strands form a native-like β-hairpin in the A state, is not correct.

## 4. Stabilization Mechanisms of Native and Non-Native Structures

### 4.1. Local Interactions

The fact that non-native helices are formed in the short fragment CHIBLΔF indicates that they are stabilized by local interactions. An interaction that possibly stabilizes the non-native helices in CHIBLΔF is the disulfide bond between Cys106 and Cys119. The CD spectrum of disulfide-reduced CHIBLΔF indicated that disulfide bond reduction significantly decreased the helical content of the fragment (Table 1). How does Cys106–Cys119 stabilize the helices of residues 98–107 and 114–135? One possibility is that the side chains of the two helices interact to stabilize the helical conformations. The presence of Cys106–Cys119 would increase the effective concentration of the interacting helical side chains, which, in turn, would increase stability. In the CHIBLΔF NOESY spectrum, however, long-range NOEs associated with such side-chain interactions were not observed [28]. A second possibility is that the entropy effect associated with helix nucleation is modulated by the disulfide bond. The helix–coil transition is approximated by the Zimm–Bragg theory [30] or Lifson–Roig theory [31]. Both theories define two parameters, namely a nucleation parameter and a propagation parameter. For nucleation, three consecutive residues must adopt helical φ–ψ angles, so that an (i,i+4) hydrogen bond can form, whereas for propagation, only one residue is required to form an additional hydrogen bond. We expect that the loop formed by Cys106–Cys119, which contains only 14 residues, should reduce the number of possible conformations allowed for these 14 residues, such that the entropic cost of nucleation is decreased compared with that required for the nucleation of an “open” chain. In CHIBLΔF, the C-terminus of the helix of residues 98–107 and the N-terminus of the helix of residues 114–135 are located within the loop formed by Cys106–Cys119. Therefore, both helices may be stabilized by the decreased unfavorable entropic cost for nucleation. However, the CD spectrum indicates that helical structures remain in the disulfide-reduced CHIBLΔF. Therefore, non-native helices are formed via not only stabilization by the disulfide bond, but also the intrinsic helical propensity of the amino acid sequence. Many peptides showed weak but detectable helicity (Table 1). The fact that the sum of the molar ellipticity values ([θ]_M_) for peptides 97–110 and 111–138 (–29.2 × 10^4^ deg·cm^2^/dmol) is close to the [θ]_M_ of the disulfide-reduced CHIBLΔF (–33.6 × 10^4^ deg·cm^2^/dmol) (Table 1), indicates that the helicity of the disulfide-reduced CHIBLΔF can be roughly explained by the intrinsic helical propensity of the amino acid sequence.

**Table 1:** Ellipticity values of peptide fragments of β-lactoglobulin at 222 nm.

Sequence	Corresponding residues	Number of Residues	[θ]_MRE _^f^ (deg·cm^2^/dmol)	[θ]_M_ × 10^–4 g^ (deg·cm^2^/dmol)	Reference
BLG	11–18	18	–6400		[12]
	(14–28)GGG(42–52) ^a^	29	–3700		[32]
	61–77	17	–5500		[12]
	85–101	16	–3300		[12]
	77–126	50	–9000/–10500		[32]
	100–126	27	–3100/–6900		[32]
	127–142	16	–6200		[12]
ELG	1–87	107 ^d^	–4800	–51.4	[33]
	88–142 (CHIBL) SS^ b^	75 ^d^	–10600	–79.3	[27]
	88–142 (CHIBL) SH^ c^	75 ^d^	–6700	–50.2	unpublished
	97–142 (CHIBLΔF) SS ^b^	54^ e^	–11900	–64.3	[28]
	97–142 (CHIBLΔF) SH ^c^	54^ e^	–6200	–33.6	[28]
	97–110	14	–5500	–7.7	[28]
	111–128	18	–4500	–8.0	[27]
	111–138	28	–7700	–21.5	[28]
	124–138 (F136Y)	15	–7200	–10.8	[27]

^a^ Sequences 14–28 and 42–52 were connected by a triglycine linker. ^b^ Cys106–Cys119 is intact. ^c^ Cys106–Cys119 is reduced. ^d^ Including the 20-residue N-terminal His tag. ^e^ Including the 8-residue C-terminal His tag. ^f^ Mean residue ellipticity. ^g^ Molar ellipticity = [θ]_MRE_ × number of residues.

### 4.2. A–C Transition

It is known that the A state of ELG is transformed to the C state by lowering the temperature [21]. A single disulfide mutant, C66A/C160A, assumes the A state in the presence of salt, but is transformed to the C state at a low ionic strength, even at room temperature. Two alternative explanations for these observations are possible. First, the native-like β-hairpin in the A state is stabilized by specific hydrophobic interactions between residues located inside and outside the CHIBL sequence. If the residues located outside CHIBL are absent, such interactions do not occur and the residues in the F- and G-strand regions cannot form the native-like β-hairpin and assume non-native helical structures based on the intrinsic helical propensity. Even if the residues located outside CHIBL are present (in the full-length ELG), hydrophobic interactions become weak at low temperature, so that the native-like β-hairpin is destabilized. Assuming that the compact globular shape in the A state is afforded by long-range hydrophobic interactions, including the interactions between residues located inside and outside the CHIBL sequence, they are weakened at low temperature, so that the molecule is transformed to the C state. At low ionic strength, electrostatic repulsion between the positive charges on the surface of the protein molecule compels the polypeptide chain to expand. Even at room temperature, therefore, the A state is transformed to the C state at low ionic strength. Because the disulfide bond Cys66–Cys160 plays a role in suppressing the expansion of the polypeptide chain, the anion-concentration-dependent A–C transition is not observed for the intact protein.

An alternative explanation is that nonspecific hydrophobic interactions cause the compact globularity observed in the A state. Generally, polymer chains collapse in a poor solvent in which the interactions between different segments of polymer chains are preferred to those between the polymer segment and solvent. This is well known as the globule–coil transition. In such a collapsed state, the native-like β-hairpin may be stabilized by the polypeptide-chain-provided environment. When the polypeptide chain is expanded at a low ionic strength or at a low temperature, the β-hairpin is destabilized and a non-native α-helix is formed via local interactions. In this case, the stabilizing interactions do not necessarily include the residues located inside the CHIBL sequence.

To discriminate between these two possibilities, the conformations of proline mutants were examined by SAXS [34]. As described above, the secondary-structure unit, including the substituted residue, was specifically destabilized by the proline substitution. If the first possibility occurs in reality, the proline mutants must become expanded. In contrast, if the second possibility occurs in reality, the proline mutants are expected to keep a compact conformation. The results of the SAXS experiment confirmed the latter case and indicated that the native-like β-hairpin was induced by the nonspecific interaction in a collapsed conformation. This was further supported by a truncation experiment [33]. The temperature-dependent or anion-concentration-dependent A–C transition was observed for full-length WT*, but not for CHIBL. Yamamoto *et al*. constructed a series of truncated proteins (30–162 and 60–162) and examined whether these proteins underwent the A–C transition [33]. If a specific interaction between residues located inside and outside the CHIBL sequence was lost by truncation, the A–C transition was expected to disappear in a truncated protein. However, the A–C transition disappeared gradually with the decrease in the chain length. This is consistent with the idea that the A state is collapsed by nonspecific interactions and that the native-like β-hairpin is stable in a collapsed state.

## 5. Role of the Non-Native Helix

The location of the non-native α-helix was clarified for a stable analogue of the burst-phase folding intermediate. To confirm directly the location in the burst-phase folding intermediate, we constructed the A123T mutant, which was designed to have a native-like tertiary structure under the native conditions and reduced helical propensity of an amino acid sequence in the region in which the non-native helix is formed in the burst-phase folding intermediate. As expected, A123T showed a native-like CD spectrum under the native conditions and a less helical intermediate in the urea-induced unfolding equilibrium and in the A state, with reduced CD intensity. Furthermore, A123T showed a reduced burst-phase CD intensity, which was in agreement with the spectrum of the A state (Okabe *et al*., submitted). These results indicate that the non-native helix is formed in a region that includes A123 (H strand) during the burst phase of the folding reaction.

In spite of the large reduction in the non-native α-helical signal, the rate constants of succeeding folding reactions of A123T were not changed significantly relative to that of WT (Okabe *et al*., submitted). This suggests that the reorganization step of the non-native α-helix does not become rate limiting. This is in contrast with results that showed that non-native interactions slow the folding of small helical proteins [35,36]. It is not surprising, however, that the breaking of non-native structure is not rate limiting for proteins of which the succeeding folding processes are intrinsically slow.

What is the role of the non-native helix in the folding of β-lactoglobulin? Free-energy landscape simulation based on a lattice model showed that the non-native helix restricts the folding route and relaxes rapid entropy reduction [37]. The simulation also suggested that all up-and-down β-barrel proteins have a tendency to form α-helices in the early stage of folding [37]. To examine this possibility, the folding reaction of human tear lipocalin (HTL) was investigated [38]. Both HTL and β-lactoglobulin belong to the lipocalin family and share the eight-stranded up-and-down β-barrel structure. However, the sequence identity between HTL and BLG or ELG is only 26% or 21%, respectively. The stopped-flow CD experiment showed that HTL did not form a non-native α-helix during the folding reaction [38]. Based on this result, the authors concluded that non-native helix formation is not general for the folding of lipocalin. The difference observed between lipocalin and β-lactoglobulin was explained by the lower helical propensity of the HTL sequence. The Cys106–Cys119 disulfide bond, which is absent in HTL, also stabilizes the non-native α-helices, as described above.

Sakurai *et al*. [39] investigated mutational effects on the folding kinetics of BLG. They prepared G17E and E44L mutants, the folding reactions of which were compared with the pseudo-wild-type BLG, C121A (both G17E and E44L contain the C121A mutation). G17E was designed to increase the helical propensity of the A strand, and E44L was prepared to increase the β-sheet propensity of the B strand. Although the burst-phase CD intensity of G17E increased, which suggests that the non-native helix formed in the A strand region was stabilized, this mutant did not assume a tertiary structure, so that the effect of non-native helix stabilization could not be evaluated. Although the burst-phase secondary structure of E44L did not differ significantly from that of C121A, its folding to the native-like conformation was retarded. Based on these results, those authors suggested that the increased β-propensity of E44L induced an incorrect β-structure and inhibited rapid folding, and that non-native helix formation around this residue might play a role in circumventing such a kinetic trap.

Conversely, it is apparent that the non-native α-helix formed in the H-strand region does not accelerate or decelerate the folding reaction of ELG, although the possibility that the non-native helix plays a role in protecting ELG against severe folding barriers, such as aggregation, remains to be examined. The formation of a non-native helix may merely reflect the fact that α-helix formation is easier than β-sheet formation. Kihara and co-workers have investigated the α-helical burst during the folding reactions of β-rich proteins, including BLG [40], ubiquitin [41], src SH3 domain [42], PI3K SH3 domain [43], Fyn SH3 domain [43], and FHA domains of Rad53 and Ki67 [44]. Those authors proposed that the α-helical burst is generalized during the folding of β-rich proteins. At present, it is plausible to consider that the formation of the non-native helix of β-lactoglobulin is also a result of the general properties of the α-helix and β-sheet conformations.
